# Associations between *CES1* variants and dosing and adverse effects in children taking methylphenidate

**DOI:** 10.3389/fped.2022.958622

**Published:** 2023-01-18

**Authors:** Jacob T. Brown, Nancy Beery, Allise Taran, Tyler Stevens, Christine Henzler, Jonathan Badalamenti, Ron Regal, Catherine A. McCarty

**Affiliations:** ^1^ University of Minnesota College of Pharmacy, Department of Pharmacy Practice and Pharmaceutical Sciences, Duluth, MN, United States; ^2^ Essentia Health Department of Pediatrics, Duluth, MN, United States; ^3^ Essentia Institute of Rural Health, Duluth, MN, United States; ^4^ Essentia Health Department of Pharmacy, Duluth, MN, United States; ^5^ University of Minnesota Supercomputing Institute, Minneapolis, MN, United States; ^6^ University of Minnesota Genomics Center, Minneapolis, MN, United States; ^7^ Department of Family Medicine and BioBehavioral Health, University of Minnesota Medical School, Duluth Campus, Duluth, MN, United States

**Keywords:** pediatrics, ADHD, methylphenidate, CES1, pharmacogenetics

## Abstract

**Background:**

Methylphenidate is the most prescribed stimulant to treat attention deficit-hyperactivity disorder (ADHD). Despite its widespread usage, a fair proportion of children are classified as non-responders to the medication. Variability in response and occurrence of adverse events with methylphenidate use may be due to several factors, including drug-drug interactions as well as pharmacogenetic differences resulting in pharmacokinetic and/or pharmacodynamic variances within the general population. The objective of this study was to analyze the effect of carboxylesterase 1 (*CES1*) variants on the frequency of adverse effects and dosing requirements of methylphenidate in children with ADHD.

**Methods:**

This was a retrospective cohort study of children and adolescents who met the inclusion criteria and had a routine visit during the enrollment period were invited to participate. Inclusion criteria included: ADHD diagnosis by a healthcare provider, between 6 and 16 years of age at the time of permission/assent, had not previously been prescribed methylphenidate, and treatment with any methylphenidate formulation for at least three consecutive months. Three months of records were reviewed in order to assess changes in dose and frequency of discontinuing methylphenidate. Participants’ ADHD symptoms, medication response, adverse effects, select vitals, and dose were extracted from the electronic health record. Saliva samples were collected by trained study coordinators. Haplotypes were assigned based on copy number in different portions of the CES1 gene. Due to limited numbers, diplotypes (combinations of two haplotypes) were grouped for analysis as CES1A1/CES1A1, CES1A1/CES1A1c and CES1A1c/CES1A1c.

**Results:**

A total of 99 participants (*n* = 30 female; *n* = 69 male) had both clinical data and *CES1* sequencing data, with an average age of 7.7 years old (range 3–15 years). The final weight-based dose in all individuals was 0.79 mg/kg/day. The most common adverse effects reported were decreased appetite (*n* = 47), weight loss (*n* = 24), and sleep problems (*n* = 19). The mean final weight-based dose by haplotype was 0.92 mg/kg for CES1A2/CES1A2, 0.81 mg/kg for CES1A2/CES1P1, and 0.78 mg/kg for CES1P1/CES1P1. After correction for multiple hypothesis testing, only one SNV, rs114119971, was significantly associated with weight-based dosing in two individuals. The individuals with the rs114119971 SNV had a significantly lower weight-based dose (0.42 mg/kg) as compared to those without (0.88 mg/kg; *p* < 0.001).

**Discussion:**

Variation in CES1 activity may impact dose requirements in children who are prescribed methylphenidate, as well as other CES1 substrates. Although intriguing, this study is limited by the retrospective nature and relatively small sample size.

## Introduction

Attention deficit hyperactivity disorder (ADHD) prevalence worldwide varies considerably, and is estimated to be around 5%, ranging from 2%–7% (1). Among children 6–17 years of age in the United States, ADHD is the most prevalent neurodevelopmental disorder, with 9.5% of all U.S. children between 6 and 17 years of age having received a diagnosis of ADHD at any point in their lifetime (2). The overall prevalence in children 3–17 years of age has increased by 33% from 1997–1999 to 2006–2008 (3). Additionally, a previously estimated cost of illness of ADHD estimated the annual individual costs of ADHD to be between $12,005 and $17,458, and the total annual societal cost between $36 and $52.4 billion (4). ADHD currently has a prevalence estimated to be around 5% (5).

Methylphenidate is first-line treatment in children and adults, is available in several formulations, and is the most prescribed stimulant to treat attention deficit-hyperactivity disorder (ADHD), as well as the most dispensed medication to adolescents 12–17 years with approximately 10 million prescriptions per year from 2002 to 2010 in children 0–17 years of age (6, 7). Despite its widespread usage, a fair proportion of children are classified as non-responders to the medication, while roughly half remain on the medication after one year (8). This variation in response may be due in part to individual pharmacokinetic differences in metabolism and/or pharmacodynamic differences in receptors and transporters. Methylphenidate is a stimulant that works by inhibiting the reuptake of norepinephrine and dopamine into presynaptic neurons, thus increasing these neurotransmitters in the brain. Due to methylphenidate's mechanism of action, research has focused on several genes looking at individual response to treatment, including the dopamine transporter, dopamine receptor, and norepinephrine transporter. Studies comparing genetic variation within these genes have led to mixed results thus far and have resulted in minimal clinical impact (9–11).

Methylphenidate is metabolized to the inactive metabolite ritalinic acid by the enzyme carboxylesterase 1 (CES1) (12). Numerous single nucleotide variants (SNVs) and copy number variants (CNVs) have been identified within the *CES1* gene, with some resulting in altered enzyme activity, and either increased or decreased levels of methylphenidate plasma concentrations at standard doses (13, 14). These differences in overall exposure may lead to differences in both clinical response and adverse effects.

Few clinical studies have been completed describing the relationship between CES1 and methylphenidate with adverse event and clinical response rates. Zhu et al. demonstrated marked pharmacokinetic differences of methylphenidate in an individual with two variants in the *CES1* gene. One variant described a nonconservative amino acid substitution of glycine to glutamic acid (G143E; rs71647871), while the second was a frameshift mutation of *CES1*. This individual experienced a markedly increased area under the curve (AUC), maximum concentration, and half-life as compared to 19 other participants, as well as greater hemodynamic increase. While the frameshift mutation is considered rare (<1%), the glycine to glutamic acid was found in 3.7%, 4.3%, and 2.0% of white, black, and Hispanic populations (13). Furthermore, the G143E variant has also been shown to impair the bioactivation of oseltamivir, which is also a substrate for CES1 (15).

Variability in response and occurrence of adverse events with methylphenidate use may be due to pharmacogenetic differences resulting in pharmacokinetic and/or pharmacodynamic variances within the general population. These differences may be the result of varying levels of drug exposure among individuals at comparable doses, which may directly impact the occurrence of adverse effects and clinical response rates. Improving treatment by selecting the most appropriate dose for an individual may lead to better utilization of ADHD medications, resulting in less treatment failure and better symptomatic control. The objective of this study was to analyze the effect of *CES1* variants on the frequency and severity of adverse effects and dosing requirements of methylphenidate in children with ADHD.

## Materials and methods

### Study design

This was a retrospective cohort study examining variation in the *CES1* gene and clinical characteristics in children and adolescents with ADHD prescribed any formulation of methylphenidate. Children and adolescents who met the inclusion criteria and had a routine visit during the enrollment period were invited to participate through mailed invitation letters and a follow-up phone call from study staff. Inclusion criteria included: ADHD diagnosis by a healthcare provider included in their medical record, between 6 and 16 years of age at the time of permission/assent, no previous trial of methylphenidate, and treatment with methylphenidate for at least three consecutive months without concomitant use of another drug to treat ADHD or MAOIs. Exclusion criteria included those with hypersensitivity to methylphenidate, marked agitation, anxiety, or tension, motor tics, diagnosis of Tourette syndrome, or any cardiac abnormality. Informed parental permission, child assent, and saliva collection were obtained at the participant's regularly scheduled appointment by trained study coordinators in cooperation with their health care provider and nursing staff. This study was reviewed and approved by the Essentia Health Institutional Review Board.

### Clinical assessment

The primary outcomes of interest included known methylphenidate adverse effects and daily dose. Participants' ADHD symptoms, medication response, adverse effects, and select vitals (height, weight, blood pressure, and heart rate) were assessed using data extracted from the electronic health record through analytics and manual abstraction conducted by research staff.

Study data were collected and managed using REDCap electronic data capture tools hosted at Essentia Health. REDCap (Research Electronic Data Capture) is a secure, web-based application designed to support data capture for research studies, providing: (1) an intuitive interface for validated data entry; (2) audit trails for tracking data manipulation and export procedures; (3) automated export procedures for seamless data downloads to common statistical packages; and (4) procedures for importing data from external sources (16, 17).

Participants and their accompanying parent or guardian were asked at the time of saliva collection to self-report race and ethnicity based on standardized data collection tools (phenxtoolkit.org) as well as the names and dates of birth of the participant's biological parents and grandparents (if available) to determine and account for relatedness of study participants at the time of analysis.

### Sample collection and CES1 sequencing

Saliva samples were collected by trained study coordinators using Oragene® DISCOVER (OGR-500 and OGR-575) saliva collection kits, and were stored in a laboratory setting until DNA extraction and gene sequencing were conducted. Each sample was extracted following the automated QIAamp DNA Mini Kit (QIAGEN, Hilden, Germany) protocol for the QIAcube (QIAGEN, Hilden, Germany). Purified DNA extracts were set to elute in 2 increments of 100 μl of AE buffer (200 μl total elution volume). DNA quantification was performed in triplicate using the Qubit™ dsDNA HS Assay Kit (Thermo-Fisher, Waltham, MA) for the 2.0 Qubit™ fluorometric quantification system according to manufacturer's instructions.

Amplicon design and approximate primer placement were performed using Geneious Primer v. 2019.2.3 against the repeat-masked CES1 locus that included 20 kbp up- and downstream (build hg38 in UCSC Genome Browser). Concurrent GC% and conserved SNP tracks were plotted in Geneious alongside the reference to facilitate visual inspection of candidate regions for primer placement such that (1) resulting amplicons had a final size of ∼6 kbp +/− 15%, and (2) amplicons overlapped by at least 200 bp. Genomic coordinates for these candidate ranges were used to iteratively design primers for each amplicon using NCBI PrimerBLAST to have a final length of 20–26 nt and calculated Tm between 58 and 62 °C. Where possible 3′ GC clamps were included and homopolymers limited to 5 nt. Oligonucleotide primers were synthesized by Integrated DNA Technologies (Coralville, IA) with standard desalting and included a single phosphorothioate linkage before the terminal 3′ base to reduce off-target amplification from primer editing (18).

Amplicons were generated using Q5 HiFi HotStart mastermix (New England Biolabs, Ipswich, MA) in a 20-µl PCR containing 10 µl 2× mastermix, 1 µl of forward primer (10 µM), 1 µl of reverse primer (10 µM), 7 µl nuclease-free water, and 1 µl (10 ng/µl) of extracted gDNA. Amplification was performed according to the following cycling conditions (all except region (4): 98 °C for 30 s, followed by 30 cycles of 98 °C for 10 s, 64 °C for 15 s, and 72 °C for 3 min, and a final extension at 72 °C for 5 min. Region 4 used an extension time of 3 min 30 s. PCR products were purified using 0.5× (v/v) Ampure XP beads (Beckman-Coulter), quantified using Picogreen, and normalized to 10 ng/µl. Normalized amplicons from the same individual were pooled at equal volume (2 µl each) and concentrated to ∼5 µl by evaporation before undergoing PacBio library creation (SMRT Bell Express Template Kit 2.0, Pacific Biosciences, Menlo Park, CA) with sample barcodes added *via* ligation. Once barcoded, all libraries were pooled together and sequenced on 1 PacBio 8M SMRT cell (3 pM loading concentration) with 2-h pre-extension.

Circular consensus reads (CCS) were generated for all samples using PacBio's ccs v.6.0.0, the default minimum of 3 full passes (three full reads through the amplicon), a minimum target length of 4,000 and a maximum target length of 8,000. Reads were aligned to the human genome (GrCh38) using pbmm2 v.1.4.0, PacBio's wrapper for minimap2 (19). Bedtools v2.29.2 (20) was used to measure coverage for both CES1 and CES1P1 (to check for off-target, non-specific amplification.) For each amplicon, 30× coverage was required for a sample to be included in the final analysis. For most amplicons, only four or fewer samples had insufficient depth to be retained in the final analysis, however amplicon 4 failed to amplify for 26 samples. There was virtually no amplification of CES1P1 (as designed). The highest coverage in any sample at any point in CES1P1 was 14× (while the same sample had an average coverage of CES1P1 of only 0.08× and a minimum of 2,654× coverage for CES1).

Variants were called from the aligned bams using freebayes v.1.3.4 (21) using the following parameters: (1) Requiring a minimum of 5 reads and 10% of reads supporting an alternate call (-C 5, -F 0.10), (2) Restricting calls to just the amplified regions, (3) “Output all alleles which pass input filters regardless of genotyping outcome or model.” (–pooled-continuous) (–haplotype-length 0), (4) Haplotype length set to 0 to emit simple SNP and indel calls (rather than complex haplotype calls) (–haplotype-length 0). Variant calls were filtered with bcftools v.1.6 (22) to remove calls with a quality score < 20. SNPs were phased using whatshap v.1.0 (23).

In a parallel analysis to the alignment and variant calling, PacBio's (long amplicon analysis tool v.2.4.2 (LAA) [https://github.com/PacificBiosciences/pblaa]) was used to collapse reads into phased haplotypes, representing distinct allele sequences for each amplicon within each sample. The relative number of reads supporting each haplotype from this analysis was used as additional confirmation of the copy number calling. Haplotypes were assigned based on copy number in different portions of the *CES1* gene, using custom R code. Due to limited numbers diplotypes (i.e., combinations of two haplotypes) were grouped for analysis as CES1A1/CES1A1, CES1A1/CES1A1c and CES1A1c/CES1A1c.

**Figure 1:**
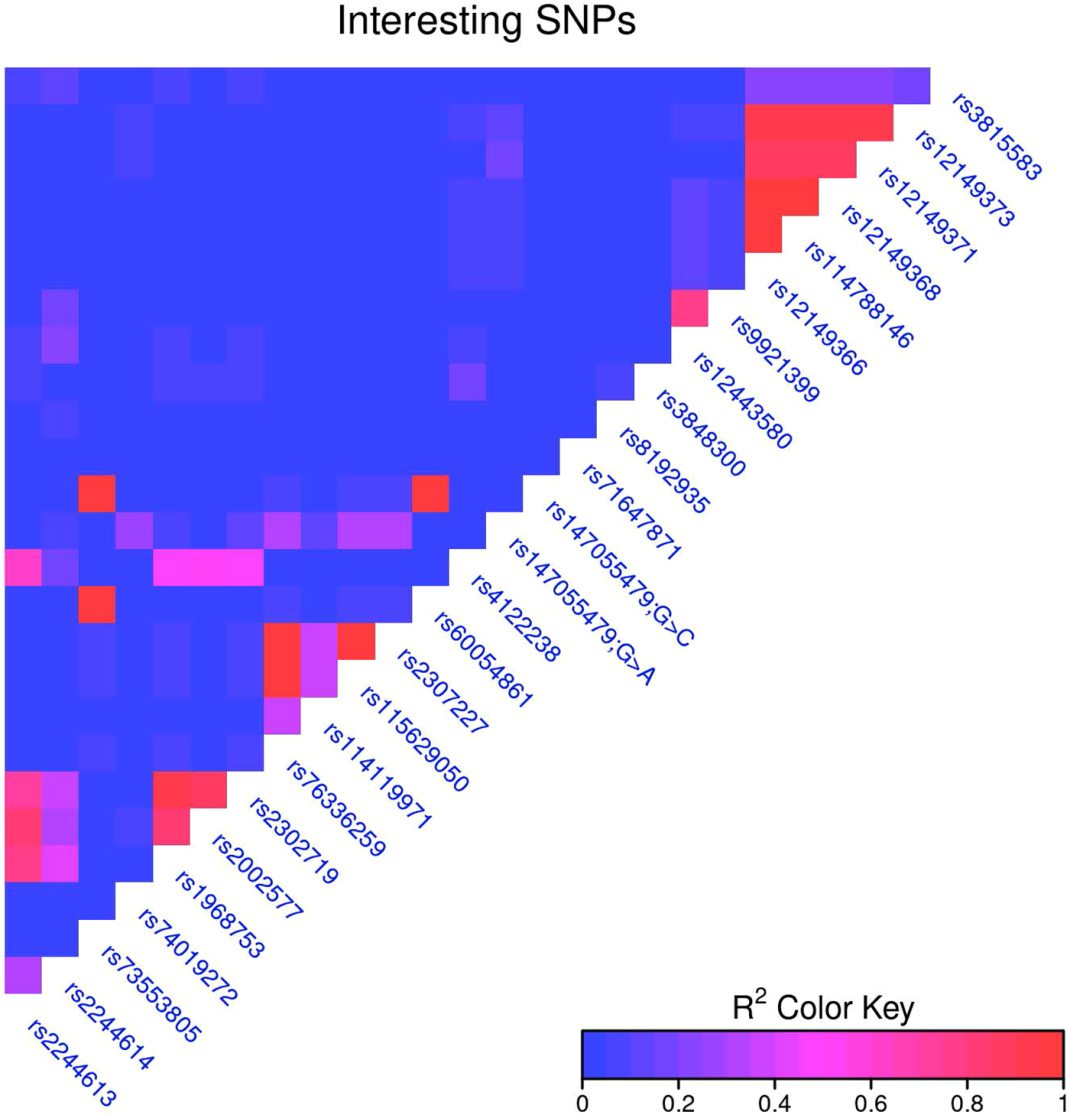
Notable CES1 SNPs.

The relationship between individual SNVs and clinical correlates were assessed for a list of SNVs determined from the literature. Linkage disequilibrium was calculated for all SNVs, and to account for the most closely linked SNVs, for every pair of SNVs with an *R*^2^ > 0.95 the SNV present in the highest number of samples was chosen for further analysis. (If two SNVs were in the same number of samples, the first one was chosen.) This dropped the number of key SNVs analyzed against clinical correlates to 19. (Supplementary File) SNVs were treated as simply present or absent, and were tested against binary clinical traits (e.g., side effects) with Fisher's exact test, and against continuous traits {e.g., log2[dose by weight (mg/kg)]} with a *t*-test. The Benjamini-Hochberg correction was used to correct for multiple hypothesis tests across all tests (all SNVs and all clinical correlates) (Figure 1).

## Results

**Figure 2:**
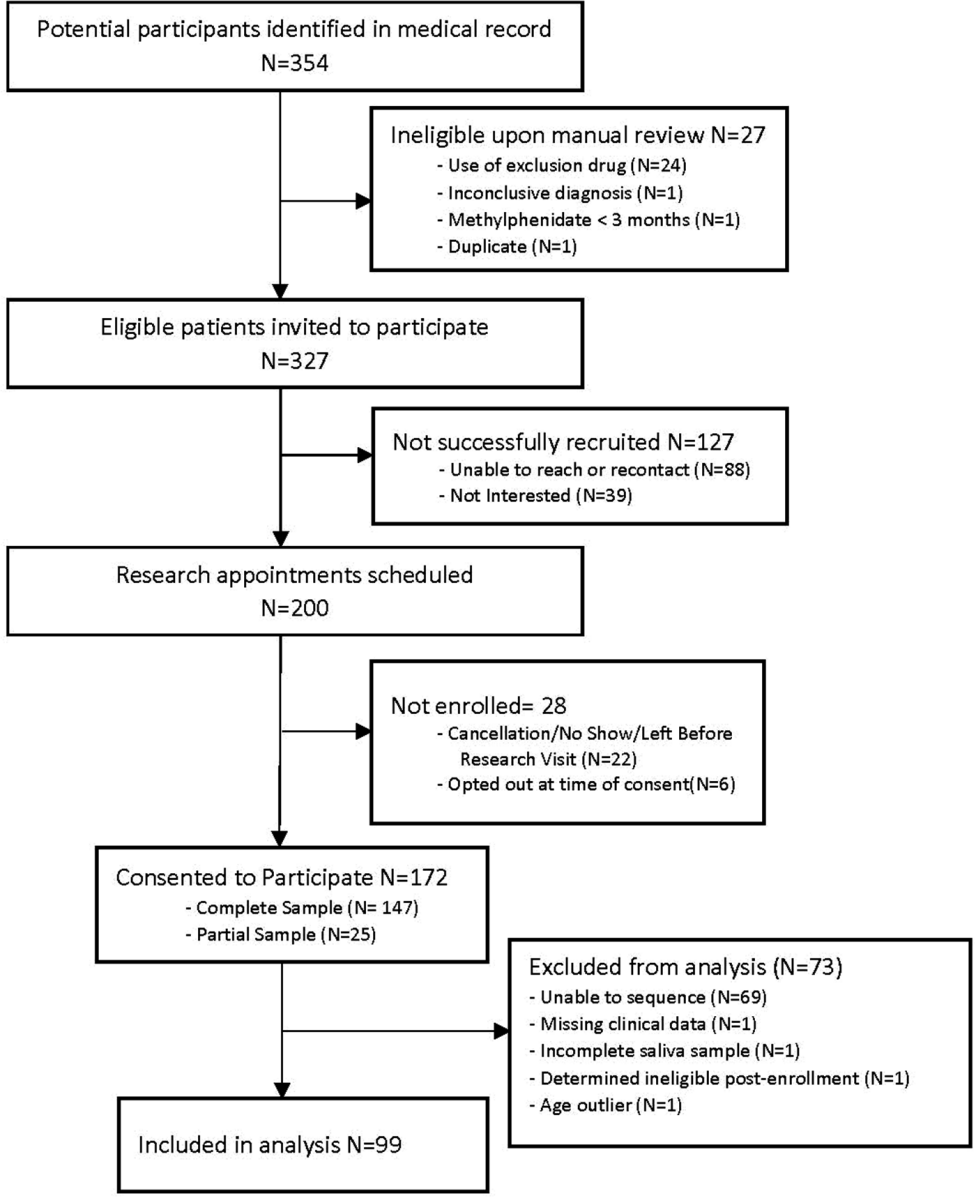
Study flowchart.

A total of 99 participants (*n* = 30 female; *n* = 69 male) had both clinical data and *CES1* sequencing data (Figure 2), with an average age of 7.7 years old (range 3–15 years) at their initial visit. Of note, methylphenidate is approved for children 6 years and older, but is sometimes used in younger children, and our data included children as young as three years of age.

**Figure 3:**
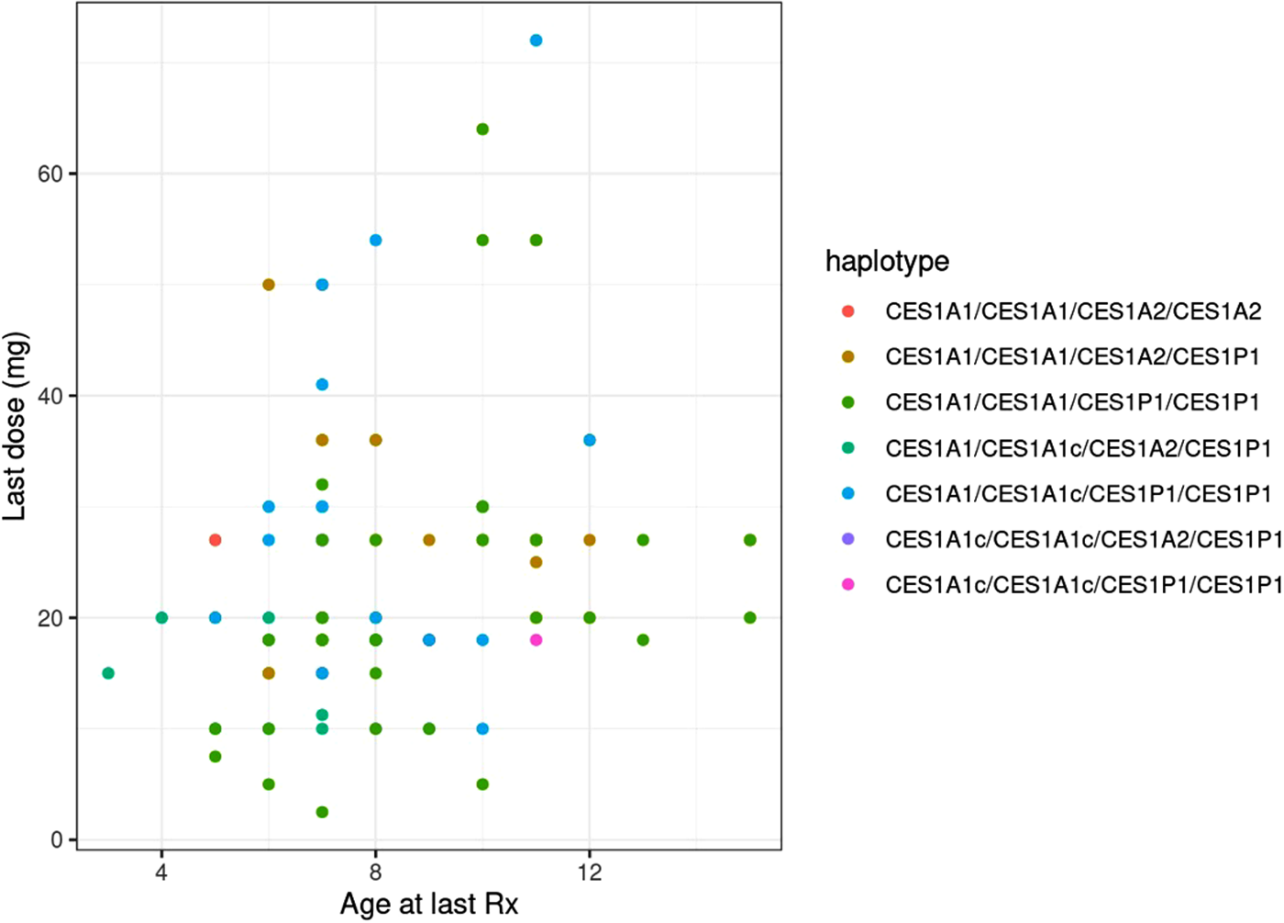
Comparing age and final methylphenidate dose across CES1 haplotypes.

Age was significantly associated with both the final absolute dose (*p* = 0.018) and weight-based dose (mg/kg; *p* = 0.005), though the *r*^2^ values for both were relatively low at 0.056 (Figure 3) and 0.078, respectively. The final average absolute dose in all participants was 24.2 mg/day (22.4 mg/day in females vs. 25.0 mg/day in males), while the final weight-based dose in all individuals was 0.79 mg/kg/day (0.74 mg/kg/day in females vs. 0.81 mg/kg/day in males). In total, 65 participants had any change in dose, 61 had a dose increase, and 15 had a dose decrease. Adverse effects were reported for 69 (female = 20, male = 49) of the individuals. The most common adverse effects reported were decreased appetite (*n* = 47; 14 females, 33 males), other (*n* = 27), weight loss (*n* = 24; 10 females, 14 males), and sleep problems (*n* = 19; 4 females, 15 males). The mean final weight-based dose by haplotype is described in Table 1.

**Table 1:** Methylphenidate dose by CES1 haplotype (mg/kg).

Haplotype	All	Males	Females
CES1A1/CES1A1/CES1A2/CES1A2 (*n* = 3)	0.92	0.92	–
CES1A1/CES1A1/CES1A2/CES1P1 (*n* = 16)	0.88	0.99	0.70
CES1A1/CES1A1/CES1P1/CES1P1 (*n* = 45)	0.75	0.77	0.69
CES1A1/CES1A1c/CES1A2/CES1P1 (*n* = 8)	0.66	0.79	0.44
CES1A1/CES1A1c/CES1P1/CES1P1 (*n* = 22)	0.90	0.88	0.94
CES1A1c/CES1A1c/CES1A2/CES1P1 (*n* = 2)	0.81	0.81	–
CES1A1c/CES1A1c/CES1P1/CES1P1 (*n* = 3)	0.38	0.38	–

The CES1 locus and 12 kb upstream were sequenced with overlapping ∼6 kb amplicons and PacBio Sequel sequencing. Nine tiled, overlapping primer sets were designed to cover all CES1 and 12 kb upstream, but primer sets 8 and 9, targeting the 3′ end of the gene, were not successful and not included in the final project. Additionally, amplicon 4 performed poorly and a subset of samples are either missing amplicon 4 or have low coverage. Amplicon 4 covers exon 2 and 3. As a result, the final sequencing data cover 12 kb upstream of CES1 to partway through intron 11 of the 14-exon CES1 gene for 99 individuals, and 19 individuals are missing data for exons 2 and 3 (amplicon 4). Most samples (77 of the 99 final samples) had no amplicons with less than the threshold 30× coverage required for further analysis, and most amplicons had vastly more than 30× coverage (median coverage for all exons was >2000×). Of the 22 samples where an amplicon was below 30× coverage, in 20 samples only one amplicon was <30×, and in the other two samples, it was two amplicons. Given the good coverage across amplicons, haplotypes could reasonably be inferred for 99 samples that also had clinical correlates (Supplementary File).

**Figure 4:**
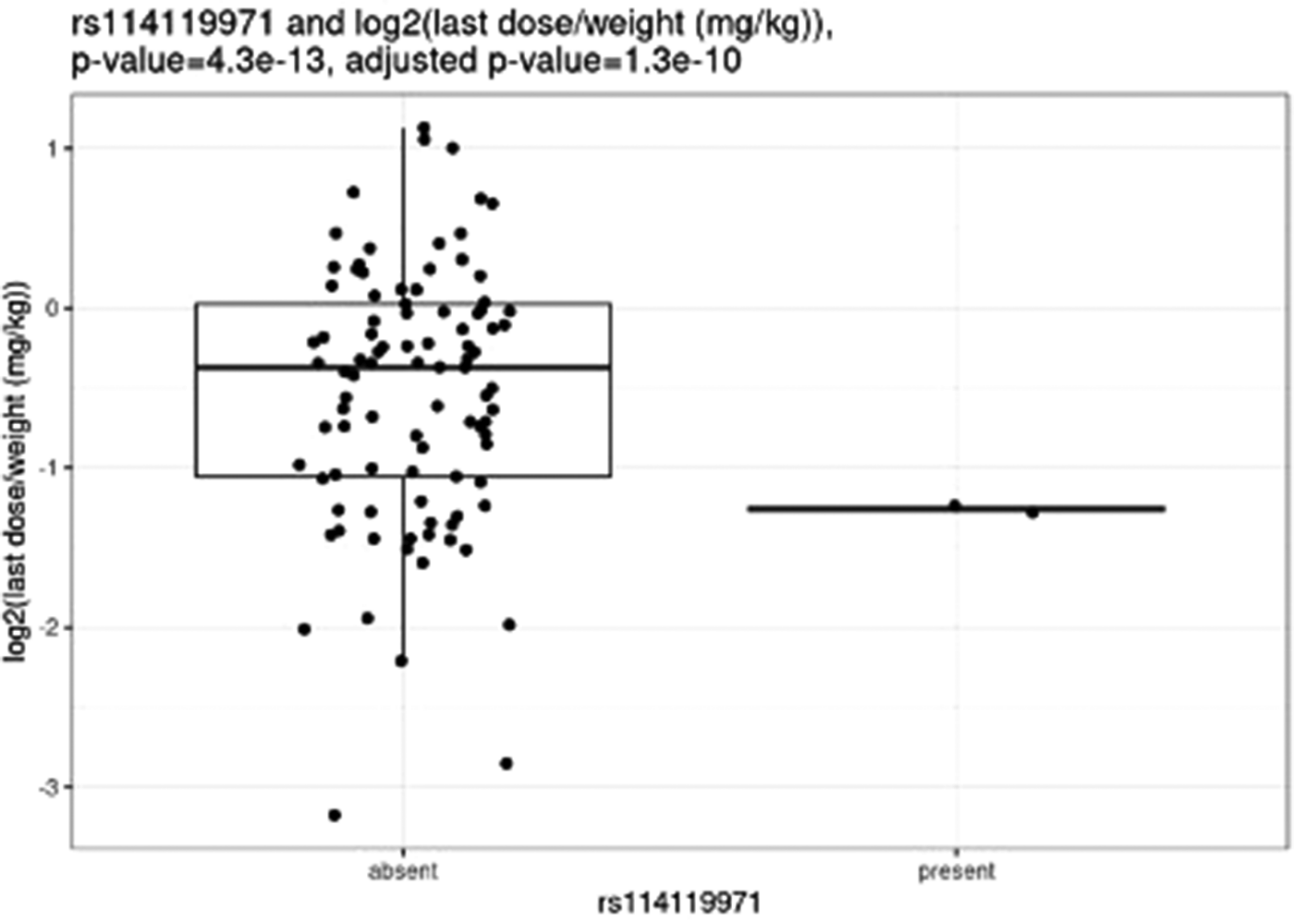
Comparing dose (mg/kg) with rs114119971.

**Figure 5:**
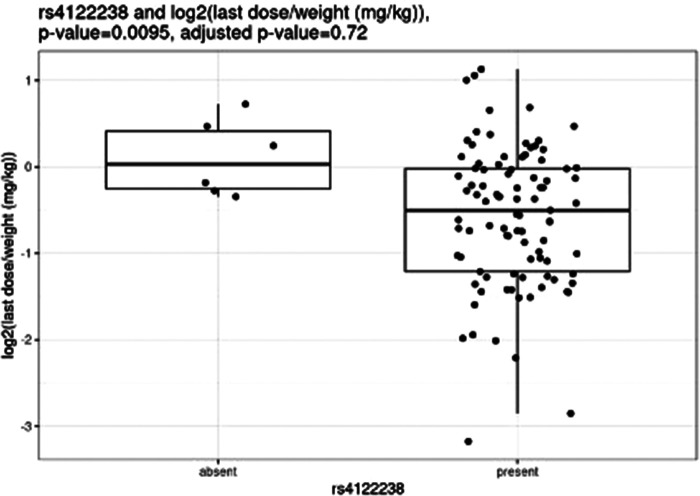
Comparing dose (mg/kg) with rs4122238.

**Figure 6:**
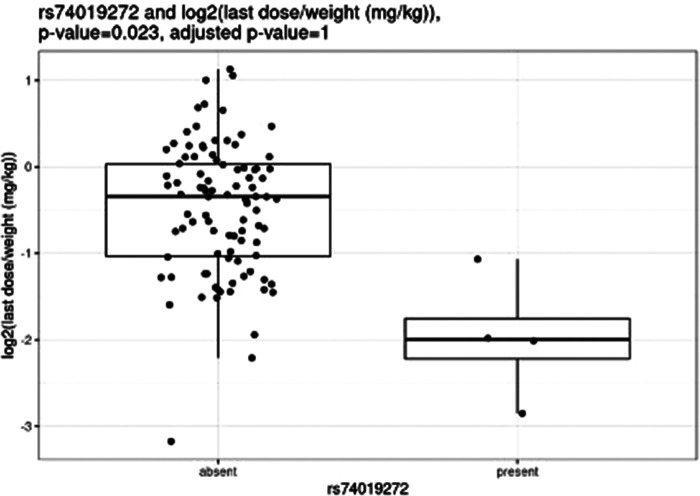
Comparing dose (mg/kg) with rs74019272.

Before correction for multiple hypothesis testing, nine SNVs showed significant associations, three with weight-based doses, five with weight loss, and one with both weight loss and dose increase. The three SNVs associated with weight-based doses [determined by t-test of log2(last dose in mg/kg) for children with and without the SNV] were rs114119971 (*p* = 4.3e–13), (Figure 4) rs4122238 (0.77 with the SNV mg/kg vs. 1.1 mg/kg without the SNV; *p* = 0.0095), (Figure 5) and rs74019272 (0.28 mg/kg with the SNV vs. 0.81 mg/kg; *p* = 0.023). (Figure 6) The six SNVs associated with weight loss were rs32171764 (*p* = 0.003), rs2244614 (*p* = 0.0039), rs1968753 (*p* = 0.0065), rs2302719 (*p* = 0.014), rs2244613 (*p* = 0.032), and rs2002577 (*p* = 0.039). SNV rs3217164 was also associated with dose increase (*p* = 0.032). Correction for multiple hypothesis testing assumes independence of tests. While the SNVs with the highest linkage disequilibrium were filtered from the dataset so only one of each pair of highly-linked SNVs was analyzed further, there was still considerable linkage disequilibrium among the remaining SNVs. As such, the remaining SNVs tested may not be truly independent, and the Benjamini-Hochberg correction for multiple hypothesis testing may be too stringent. It is worth considering the tests that were still significant after multiple hypothesis correction as the strongest result, but those that were only significant prior to the correction may also be worth consideration for further research.

After correction for multiple hypothesis testing, only one SNV, the previously mentioned rs114119971, was significantly associated with weight-based dosing, and included only two individuals with the SNV. The two individuals with the rs114119971 SNV had a significantly lower weight-based dose [as determined by a t-test of log2(final dose in mg/kg) in children with and without the SNV, final dose with rs114119971 = 0.42 mg/kg] as compared to those without the SNV (final dose = 0.88 mg/kg; *p* = 4.3e–13, adjusted *p* = 1.3e–10). One individual was identified as having the rs71647871 SNV, with a final absolute dose of 27 mg and weight-based of 0.37 mg/kg.

## Discussion

Due to the many variables involved, clinical implementation of pharmacogenetic testing in children with ADHD has proven challenging. Although this study was unable to discern any associations between clinical response to methylphenidate and *CES1* genetic variation, we did identify several *CES1* SNVs associated with weight-based dosing and weight loss; however, after correction for multiple hypothesis testing only a single SNV remained statistically significant. While sparsely studied, associations between *CES1* and methylphenidate dose, response, and adverse effects have been previously reported.

Previously, pharmacokinetic studies have described a handful of variants significantly associated with methylphenidate exposure. The previously noted Zhu et al. article described two functional *CES1* variants in a single adult resulting in decreased enzyme activity as shown by increased exposure to methylphenidate (13). Additionally, Stage et al. showed a similar increased exposure to methylphenidate of the rs71647871 SNV in a group of Danish adults (14). Similar effects for the rs71647871 variant were shown when examined as the metabolic ratio of ritalinic acid to methylphenidate (24). While the pharmacokinetic impact of this variant is well defined, only a single individual with this variant was included in this study.

Nemoda et al. described children classified as responders to methylphenidate with the glycine to glutamic variant allele require significantly smaller dosing requirements as compared to responders without the variant allele (0.41 vs. 0.57 mg/kg) (25). However, these results are limited as they were based upon five individuals with the variant allele. We were unable to compare our own results to these, as only one individual completing all study procedures also had this variant. Of the variants with significant associations with weight-based dosing, the rs114119971 SNP is a missense variant, while the rs4122238 and rs74019272 are both intron variants.

The rs114119971 SNP identified as being significantly associated with weight-based dosing after multiple hypothesis correction is a missense variant with a low allele frequency (0.2%–0.9%). No other studies were identified describing this variant with methylphenidate (or any other CES1 substrate) dosing or clinical response, and this SNP was found in only two participants in the study described herein. Of note, these two individuals required significantly lower doses as compared to those without the rs114119971 variant. Further study examining the pharmacokinetic effect of this variant on methylphenidate and other CES1 substrates may be warranted.

Recent clinical studies have compared side effect profile with genetic variation in the *CES1* gene in children taking methylphenidate for ADHD. Specifically, Johnson et al. identified two *CES1* SNV markers (rs2244613 and rs2002577) in linkage disequilibrium with two SNVs in the norepinephrine transporter gene associated with sadness as a side effect (26). While our study did not find an association between these SNPs and sadness, they were found to be associated with weight loss prior to multiple hypothesis correction. The rs2244613 variant has also been associated with low trough concentrations of dabigatran, which is also a CES1 substrate requiring bioactivation, with an allele frequency of approximately 13.2%–40.3% (27). Additionally, Bruxel et al. described a different *CES1* variant (rs3815583) where carriers had a significant odds ratio of 3.5 for appetite reduction worsening as compared to those who lacked the variant (28), although we did not find a similar association.

Of the five SNPs associated with weight loss, all were intron variants with high allele frequencies. The rs2244614 variant has an allele frequency of 18%–50% and was not shown to impact the pharmacokinetics or toxicity of capecitabine in colorectal cancer patients (29). An additional study examining the pharmacodynamic effects of CES1 variation, including rs2244614, in patients treated with angiotensin converting enzyme (ACE) inhibitors with congestive heart failure did not show significant associations (30). The rs1968753 variant has an allele frequency of 19%–45% and was previously shown to possibly be a candidate for risk prediction of antituberculosis drug-induced hepatotoxicity (31). The rs2244613 variant is relatively well described in CES1 substrates other than methylphenidate and has an allele frequency of 15%–40%. The rs2244613 variant has been associated with lower dabigatran trough concentrations (27), reduced risk of diarrhea with irinotecan treatment (32), and lower peak and trough enalaprilat concentrations (33). The rs2302719 and rs2002577 SNVs are both intron variants with relatively high allele frequencies, though no studies were identified describing these.

While this study focused on the impact of variation in CES1 on methylphenidate dosing and adverse effects, other non-genetic factors also contribute to CES1 activity. These include natural products capable of inhibiting CES1 activity (34), alcohol (35), and therapeutic agents which may result in drug-drug interactions (36). Additionally, variation in several other genes have been described as it relates to methylphenidate adverse events and clinical response (37, 38).

Although studies describing the pharmacokinetic and pharmacodynamic effects of CES1 on medications such as methylphenidate, ACE-inhibitors, and dabigatran are increasing, CES1 is rarely included in commercially available pharmacogenetic testing panels. Based on registered laboratories through the National Library of Medicine Genetic Testing Registry, only six laboratories include *CES1* genetic testing, five of which are located within the United States. Variants such as rs71647871 that have been shown to increase exposure to CES1 substrates could be clinically useful in identifying individuals who may respond better to a lower dose or an alternative medication.

This study is limited by the retrospective nature of the clinical and adverse effect data, and is dependent on medical records. It is also limited by the inclusion of all methylphenidate formulations considered as the total daily dose of methylphenidate. Additionally, given the considerable variation of *CES1* the sample size is relatively small to show clinically significant differences in dosing or adverse effects.

Contributions of this study include improving researchers' and clinicians' understanding of how genetic variation in the *CES1* gene primarily responsible for the metabolism of methylphenidate impacts adverse events and dose requirements in children with ADHD.

## Data Availability

The original contributions presented in the study are publicly available. This data can be found here: https://www.ncbi.nlm.nih.gov/bioproject/PRJNA919062.
