# Supplementary material for: Associations between CES1 variants and dosing and adverse effects in children taking methylphenidate
Source: Front Pediatr. 2023 Jan 18;10:958622. doi: 10.3389/fped.2022.958622 (PMC9890192; doi:10.3389/fped.2022.958622)
Supplement: Supplementary file 1 [file Datasheet1.docx]

**Assignment of CES1/*CES1P1* haplotypes**

CES1 gene structure is complicated by the presence of *CES1P1* (sometimes called CES1A3), a six-exon non-functional pseudogene found just upstream of CES1 on chr16, and in the opposite orientation (CES1 is on the negative strand, *CES1P1* is on the positive strand). At the *CES1P1* locus, instead of the canonical *CES1P1* sequence, some individuals have CES1A2, a variant of CES1 that has exon 1 and some sequence upstream (5’UTR) and downstream (into intron 1) from *CES1P1*, but the rest of the gene is identical CES1. There are several described CES1 haplotypes, encompassing both CES1 and *CES1P1* (Stage et al. 2017). Here, following Stage et al. 2017, we distinguish two forms of CES1: CES1A1 and CES1A1c, (where CES1A1 is the canonical CES1 sequence, and CES1A1c has CES1P1’s exon 1), and two forms of CES1P1: CES1P1 (aka CES1A3) and CES1A2.

#### **Detecting CES1A1c**

Amplicon 3 covers exon 1 of CES1. The primers, however, are located far enough upstream (3635 bp) and downstream (2768 bp) from the exon itself, that even with the gene conversion to the CES1P1 exon 1 that we see in CES1A2, the primers will not amplify CES1A2 (because the upstream sequence for CES1A2 is CES1P1, which the primers won’t anneal to). The CES1A1c allele, which also has conversion of exon 1 to the CES1P1 exon 1, but in the genomic context of CES1 (i.e. upstream and downstream sequences are CES1) should be amplified by the amplicon 3 primers just as well as CES1A1. However, CES1A1c amplicon 3 sequences should align to CES1, but show variants in exon 1 that correspond to differences between exon 1 of CES1P1 and exon 1 of CES1A1.

The difference between the CES1P1 RefSeq sequence in this region (for exon 1 and 3kb up/downstream) compared to CES1 was analyzed using blat (Kent 2002). Inspection of the results showed 9 variants (all SNPs) between the CES1P1 reference sequence and the CES1 reference sequence for exon 1 (including 5’ UTR). Further examination showed 7 variants (1 deletion, 6 SNPs) between the CES1P1 reference sequence and the CES1 reference sequence within 200 bp upstream of the start of the CES1 5’ UTR, and 16 variants (1 insertion, 15 SNPs) within 200 bp downstream of the CES1 exon (into the CES1 intron 1).

In the data, there were 14 variants within 200bp of exon 1 (including exon 1 and the 5’ UTR), and 12 were part of the 32 differences between the CES1P1 reference sequence and the CES1 reference sequence. Of these, seven SNPs occurred together (centered on exon 1 with 5 SNPs in the exon and one each in the 5’ UTR and exon 1) and were used to define a CES1A1c haplotype.

Using these seven SNPs to define CES1A1c, there were 30 samples with one copy of CES1A1c (i.e heterozygous at these sites), 5 samples with two copies of CES1A1c (i.e. homozygous at these site), and 64 samples with no copies of CES1A1c.

**Amplicons**

The CES1 locus and 12kb upstream were sequenced with overlapping ~6kb amplicons and PacBio sequencing, and CES1 haplotypes were inferred from the resulting variant allele frequencies. Determining CES1 haplotypes is challenging using amplicon data because the primers in this study are CES1-specific (don’t amplify *CES1P1*), and amplicons preclude read-depth copy number approaches. As a result, copy numbers were assessed from variant allele frequencies as follows.

#### **Amplicon-level BAF calculation**

Allele frequencies were calculated for all SNPs. The allele frequency should be consistent across each amplicon, since if a given primer set amplifies X alleles (0,1,2,3 or more), it amplifies those allele(s) across the whole amplicon consistently. For each amplicon in CES1, the mean allele frequency was calculated, excluding uninformative sites, defined as variants with allele frequencies >90% or <10%. To simplify the subsequent analysis and visualization, all variant allele frequencies were normalized by subtracting all allele frequencies >50% from 1 (i.e. 75% was changed to 25%).

#### **Calling haplotypes**

Amplicon 3, as discussed above in the *Detecting CES1Ac* section, amplifies only the alleles of CES1 (CES1A1 or CES1A1c). Amplicons 1 and 2 are upstream of amplicon 3, and likewise only amplify CES1 alleles, not CES1A2 or CES1P1 (CES1A3). Amplicon 4 performed poorly in 22 samples, and therefore was not used for copy number assessment. Amplicons 5-7 amplify sequence from CES1A1, CES1A1c and CES1A2 equally well, allowing copy number and therefore haplotype to be inferred from the variant allele frequencies at these three amplicons. Therefore, haplotypes were called based on the median variant allele frequency for each amplicon for amplicons 5, 6 and 7. A sample was called as two copies (expected frequency of 0.5) if the variant allele frequency was >= 0.4, called as 3 copies (expected normalized frequency of 0.33), if it was <0.4 but greater than 0.29, and called as 4 copies (expected normalized frequency of 0.25) if it was <=0.29. All samples where copy number was ambiguous were not considered for further analysis, limiting the dataset to 99 samples.

Due to low sample numbers for each of the possible diplotypes (combinations of two haplotypes), we collapsed the CES1A1/CES1A1, CES1A1/CES1A1c and CES1A1c/CES1A1c diplotypes into one group.

#### **Linkage disequilbrium**

There were 52 SNPs identified from the literature (Marsh et al. 2004, Wang et al. 2017, Zhu 2008, Bruxel 2013, Johnson e 2013 and PharmGKB) for further analysis, 26 of which were found in this dataset. Six of the 26 SNPs were removed because they were highly linked (R^2^ > 0.95) with another SNP in this dataset that had more samples. (If two SNPs were in the same number of samples, the first one was chosen.) One additional SNP, rs71647871 was found in only one sample, so was removed from further analysis. (This is G143E, which is known to affect methylphenidate metabolism.) This left 19 SNPs for further analysis.

Table of the final SNPs assessed in this study:

| Chr | position | ref | alt | rsid | Exonic/  intronic | exon | mutation type | amplicon | N (of 99) |
| --- | --- | --- | --- | --- | --- | --- | --- | --- | --- |
| 16 | 55810697 | G | T | rs2244613 | intronic | . | . | CES1_amp7 | 93 |
| 16 | 55810705 | G | A | rs2244614 | intronic | . | . | CES1_amp7 | 77 |
| 16 | 55810828 | G | A | rs73553805 | intronic | . | . | CES1_amp7 | 2 |
| 16 | 55810891 | G | A | rs74019272 | intronic | . | . | CES1_amp7 | 4 |
| 16 | 55811439 | A | G | rs1968753 | intronic | . | . | CES1_amp7 | 90 |
| 16 | 55815590 | G | C | rs2002577 | intronic | . | . | CES1_amp6 | 90 |
| 16 | 55816796 | T | G | rs2302719 | intronic | . | . | CES1_amp6 | 89 |
| 16 | 55816819 | G | A | rs76336259 | intronic | . | . | CES1_amp6 | 5 |
| 16 | 55819586 | G | C | rs114119971 | exonic | exon 7 | nonsynonymous SNV | CES1_amp6 | 2 |
| 16 | 55822805 | A | G | rs4122238 | intronic | . | . | CES1_amp5 | 93 |
| 16 | 55823540 | G | A | rs147055479 | intronic | . | . | CES1_amp5 | 13 |
| 16 | 55827882 | A | G | rs8192935 | intronic | . | . | CES1_amp4 | 9 |
| 16 | 55828693 | G | A | rs3848300 | intronic | . | . | CES1_amp4 | 55 |
| 16 | 55832425 | T | C | rs12443580 | intronic | . | . | CES1_amp3 | 60 |
| 16 | 55832466 | T | C | rs9921399 | intronic | . | . | CES1_amp3 | 52 |
| 16 | 55833022 | A | C | rs12149366 | exonic | exon 1 | nonsynonymous SNV | CES1_amp3 | 35 |
| 16 | 55833094 | T | C | rs12149371 | UTR5 | . | . | CES1_amp3 | 39 |
| 16 | 55833101 | T | C | rs12149373 | upstream | . | . | CES1_amp3 | 37 |
| 16 | 55833130 | A | C | rs3815583 | upstream | . | . | CES1_amp3 | 38 |

The seven SNPs removed from analysis because they were too tightly linked to other SNPs or were only in one sample were:

| Chr | position | ref | alt | rsid | Exonic/  intronic | exon | mutation type | amplicon | N (of 99) |
| --- | --- | --- | --- | --- | --- | --- | --- | --- | --- |
| 16 | 55819633 | C | A | rs115629050 | exonic | exon 7 | nonsynonymous SNV | CES1_amp6 | 5 |
| 16 | 55821449 | G | T | rs2307227 | exonic | exon 5 | nonsynonymous SNV | CES1_amp5 | 5 |
| 16 | 55821501 | C | G | rs60054861 | exonic | exon 5 | nonsynonymous SNV | CES1_amp5 | 2 |
| 16 | 55823540 | G | A | rs147055479 | intronic | . |  | CES1_amp5 | 2 |
| 16 | 55823658 | C | T | rs71647871 | exonic | exon 4 | nonsynonymous SNV | CES1_amp5 | 1 |
| 16 | 55833037 | T | C | rs114788146 | exonic | exon 1 | nonsynonymous SNV | CES1_amp3 | 35 |
| 16 | 55833057 | G | C | rs12149368 | UTR5 | . | . | CES1_amp3 | 35 |

**References**

Kent WJ. BLAT - the BLAST-like alignment tool. Genome Res. 2002;12(4):656-64.

Stage C, Jürgens G, Guski LS, Thomsen R, Bjerre D, Ferrero-Miliani L, et al. The impact of CES1 genotypes on the pharmacokinetics of methylphenidate in healthy Danish subjects. Br J Clin Pharmacol. 2017;83(7):1506–14.
